# Pharmacokinetic Analysis of Carnosic Acid and Carnosol in Standardized Rosemary Extract and the Effect on the Disease Activity Index of DSS-Induced Colitis

**DOI:** 10.3390/nu13030773

**Published:** 2021-02-27

**Authors:** Jacob P. Veenstra, Bhaskar Vemu, Restituto Tocmo, Mirielle C. Nauman, Jeremy J. Johnson

**Affiliations:** Department of Pharmacy Practice, College of Pharmacy, University of Illinois at Chicago, Chicago, IL 60612, USA; jveens2@uic.edu (J.P.V.); vemu@uic.edu (B.V.); rttocmo@uic.edu (R.T.); mnauma6@uic.edu (M.C.N.)

**Keywords:** disease activity index (DAI), inflammatory bowel disease (IBD), rosemary, sestrin 2, tight junctions

## Abstract

Rosemary extract (RE) is an approved food preservative in the European Union and contains dietary phytochemicals that are beneficial for gastrointestinal health. This study investigated the effects of RE on dextran sodium sulfate (DSS)-induced colitis and also determined the pharmacokinetics of dietary phytochemicals administered to mice via oral gavage. Individual components of rosemary extract were separated and identified by LC–MS/MS. The pharmacokinetics of two major diterpenes from RE, carnosic acid (CA) and carnosol (CL), administered to mice via oral gavage were determined. Then, the effect of RE pre-treatment on the disease activity index (DAI) of DSS-induced colitis in mice was investigated. The study determined that 100 mg/kg RE significantly improved DAI in DSS-induced colitis compared to negative control. Sestrin 2 protein expression, which increased with DSS exposure, was reduced with RE treatment. Intestinal barrier integrity was also shown to improve via fluorescein isothiocyanate (FITC)–dextran administration and Western blot of zonula occludens-1 (ZO-1), a tight junction protein. Rosemary extract was able to improve the DAI of DSS-induced colitis in mice at a daily dose of 100 mg/kg and showed improvement in the intestinal barrier integrity. This study suggests that RE can be an effective preventative agent against IBD.

## 1. Introduction

Inflammatory bowel disease (IBD) is characterized by chronic inflammation of the gastrointestinal (GI) system and affects more than 3.1 million people in the United States and 6.8 million globally [1,2]. The two most common types of IBD include ulcerative colitis (UC) and Crohn’s disease (CD). While UC affects mainly the colon, CD can occur anywhere along the GI tract [3]. The pathogenesis of IBD involves several risk factors including genetic susceptibility, diet, and the diversity of the gut microbiota [4,5]. In most cases, the development of IBD is accompanied by weakened intercellular connections between the cells in the intestinal epithelium, known as tight junctions (TJs), due to genetic factors, unhealthy diet, and changes in gut microbiota [6,7]. Weakening of the intestinal barrier integrity allows for infiltration of bacterial and dietary antigens into the lamina propria, triggering an immune response involving the recruitment of T cells, dendritic cells, and macrophages [8,9,10]. This response initiates a cascade of cytokines that promote inflammation and reactive oxygen species (ROS) signaling [11]. An immune response that remains unresolved may promote chronic inflammation leading to IBD development.

Given the complexity and involvement of multiple signaling pathways that contribute to the etiology of inflammatory conditions of the GI tract, there is a need to identify foods and phytochemicals that can modulate cell signaling pathways and novel targets that can ameliorate IBD. Rosemary (*Salvia rosmarinus*, formerly known by the scientific name *Rosmarinus officinalis*) is an herb native to the Mediterranean region and has been used in traditional medicine for its analgesic, anti-nausea, memory improvement, and immune-boosting properties [12,13,14]. Research has shown that rosemary possesses anti-oxidant, anti-bacterial, anti-inflammatory, and anti-cancer properties, suggesting it could be a potential treatment for several diseases [15,16]. Rosemary is also an approved food preservative in the European Union and is a Generally Recognized as Safe (GRAS) substance in the United States [13,17]. Depending on the preparation of the extract, an oil-soluble rosemary extract (RE) is most often reported to be rich in diterpenes, most notably carnosol (CL) and carnosic acid (CA), which are phenolic diterpenes with potent anti-oxidant capabilities that show potential as anti-cancer agents [12]. Studies have shown that CL modulates the activity of several proteins including AMP-activate protein kinase (AMPK), nuclear factor erythroid 2-related factor 2 (Nrf2), sestrin 2, and mechanistic target of rapamycin (mTOR) [18,19].

Sestrins, which are a family of stress-inducible proteins that regulate cellular homeostasis and survival mechanisms in oxidative stress, apoptosis, and autophagy, are a promising target for IBD treatment [20,21,22]. Sestrins possess anti-oxidant functionality and also closely regulate autophagy via mTOR inhibition [23,24]. Additionally, sestrins are potential therapeutic targets against diseases such as diabetes, cardiac disease, neurodegenerative disease, and cancer [25,26,27]. Sestrin 2 in particular has been identified as an important regulator of oxidative stress and apoptosis. Two major pathways that induce sestrin 2 expression are Nrf2-Keap1 and the unfolded protein response (UPR) pathways, which are induced through oxidative stress and accumulation of misfolded or damaged proteins, respectively [28]. Additionally, sestrin 2 downregulates the mTOR activity, which is a strategy for treating colitis in humans due to its role in regulating cellular stress and inflammation [29]. Several studies have shown the effectiveness of mTOR inhibitors in colitis treatment [30,31,32]. Although these functions of sestrin 2 are well established, its role in IBD development is poorly understood. Clinically, sestrin 2 has been shown to be elevated in patients with ulcerative colitis [33]. The same study also showed that sestrin 2-/- mice could not recover from dextran sodium sulfate (DSS)-induced colitis. Furthermore, low sestrin 2 expression correlates with colitis-associated colorectal cancer incidence, possibly due to loss of p53 activity [34]. These findings suggest that sestrin 2 protein expression can be indicative of disease state and may follow a nonlinear response pattern depending on the type of disease. In one instance low sestrin 2 expression appears to be beneficial for colitis, while elevated expression of sestrin 2 appears to be beneficial for colon cancer.

Recently, RE and its phytochemical constituents have been evaluated as possible treatments for IBD in DSS and trinitrobenzene sulfonic acid (TNBS)-induced colitis mouse models [35,36]. Our hypothesis was that RE extract modulates Nrf2 activity and sestrin 2 protein expression, resulting in improved disease activity index (DAI) in mice with DSS-induced acute colitis. The goal of this study was to determine the ability of RE to prevent colitis development in vivo and to determine the mechanism of colitis prevention. In this study, an oil-soluble extract from rosemary leaves was evaluated by semi-preparative HPLC, and its major components were identified by LC–MS. The pharmacokinetics of CL and CA from RE administered orally to mice were also determined. A DSS model of colitis was used to investigate the ability of RE to prevent colitis induction and to evaluate regulation of TJ protein expression. DAI was used to quantitate the extent of disease and was scored on the basis of the following parameters: percent change in body weight, fecal consistency, fecal blood, and spleen weight (g). The results suggest that rosemary pre-treatment improves the DAI of mice compared to colitis mice and modulates protein targets important to regulating inflammation and oxidative stress.

## 2. Materials and Methods

### 2.1. Chemicals and Reagents

Commercially available RE was purchased from Vitiva (Markovci, Slovenia). CA and CL were purchased from Cayman Chemicals (Ann Arbor, MI, USA). Acetonitrile and formic acid were purchased from Thermo Fisher Scientific (Waltham, MA, USA). Butyl paraben was purchased from Acros Organics (Fair Lawn, NJ, USA). Mouse plasma was purchased from Innovative Research Inc. (Novi, MI, USA). Dextran sodium sulfate was purchased from Alfa Aesar (Haverhill, MA, USA). RNAlater was purchased from Thermo Fisher Scientific (Waltham, MA, USA). Fluorescein isothiocyanate (FITC)–dextran (40 kDa) was purchased from Sigma-Aldrich (St. Louis, MO, USA). Sestrin 2, zonula occludens-1 (ZO-1), and glyceraldehyde-3-phosphate dehydrogenase (GAPDH) rabbit polyclonal primary antibodies were purchased from Proteintech (Rosemont, IL, USA). Horseradish peroxidase (HRP)-linked anti-rabbit secondary antibodies were purchased from Cell Signaling Technology (Danvers, MA, USA).

### 2.2. Identification of Major Components in Rosemary Extract

Dried RE (2 g) were extracted 3 times with 10 mL of methanol at room temperature for 2 h each. Samples collected from the organic fraction were analyzed on a HPLC Ultra Fast Liquid Chromatography (UFLC) system equipped with a photodiode array detector and a Phenomenex Kinetex C18 column (250 mm × 4.60 mm i.d., 5 μm; Torrance, CA, USA). The elution conditions were as follows: flow rate, 0.5 mL/min; column temperature, 30 °C; injection volume, 5 µL; detector wavelength, 280 nm. The mobile phases consisted of (A) 70% deionized water, 30% acetonitrile, and 0.1% formic acid; (B) 40% water, 60% acetonitrile, and 0.1% formic acid. Optimal gradient elution was as follows: 100% A for the initial 4 min, and then from 4 to 34 min the composition was ramped gradually to 100% solvent B. From 34 to 45 min, the eluent composition was maintained at 100% solvent B, and from 45 to 50 min the eluent composition was returned to 100% solvent A. Peak identification was performed by comparing elution times to commercially available standards. Other major peaks with no commercially available standards were identified by isolating pure compounds. Semi-preparative isolation was performed on a Pure Chromatography System (Buchi Corporation, New Haven, DE, USA). Dried RE (50 g) was subjected to a scaled-up methanolic extraction as described above. Separation was conducted on a 25 cm × 21.2 mm, 5 μm Discovery C18 column (Supelco, Bellefonte, PA, USA). Elution gradient was the same as described above. Pooled highly pure (>85%) fractions were dried on a rotary evaporator (Buchi Corporation, New Haven, DE), dissolved in an HPLC-grade methanol, and subjected to electrospray ionization–mass spectrometry (negative ion mode) via direct infusion. Mass spectra obtained were analyzed and/or compared to previous reports to confirm the identities of compounds [37,38,39].

### 2.3. Animals and Treatment

Male C57BL/6 mice, aged 5 to 6 weeks, were obtained from Jackson Labs (Bar Harbor, ME, USA). The mice were housed in plastic cages and given standard chow and water ad libitum. Mice were acclimatized for 1 week on a 12 h light/dark cycle before experiments. All experiments were performed according to the policies and standards of the Institutional Animal Care and Use Committee (IACUC) of University of Illinois at Chicago. All care was taken to minimize suffering to the animals.

### 2.4. Method Development for Carnosic Acid and Carnosol Detection

Analytical standards of either CA or CL were prepared at concentrations ranging from 0 to 10 mg/mL in acetonitrile (ACN). Butyl paraben (BP) was used as the internal standard. Mouse plasma was spiked with the standards and added to ice-cold ACN containing 0.1% formic acid and 0.5 mg/mL BP on ice to promote deprotonation. The mixture was then vortexed and centrifuged at 14,000× *g* for 15 min to remove precipitates from the plasma. The supernatant was transferred to a vial for LC–MS/MS sampling. A gradient elution with a flow rate of 0.35 mL/min was used. Mobile phase A was water with 0.1% formic acid, and mobile phase B was acetonitrile with 0.1% formic acid. The gradient was as follows: 95% solvent A was used for the initial 0.5 min, followed by a gradual change to 95% B from 0.5 to 2 min and holding at 95% B from 2 to 6 min. Elution then gradually changed to 95% A from 6 to 6.1 min and remained at 95% A from 6.1 to 10 min. A Sciex QTRAP 3200 MS/MS (Redwood City, CA, USA) was used to detect compounds of interest.

### 2.5. Rosemary Extract Pharmacokinetic Dosing and Analysis

Pharmacokinetic analysis of CA and CL from RE was performed in C57BL/6 mice (permission number 19-200). A total of 32 mice were divided into 2 separate groups. Dried RE was dissolved in cottonseed oil, and mice were administered a single dose of 100 mg/kg RE via oral gavage, which resulted in a human equivalent dose (HED) of 8.1 mg/kg. This dose was considered safe on the basis of previous studies from our lab that had no adverse effects [40]. Mice were dosed at 8 separate time points ranging from 0.25 to 24 h. After the set time, blood was drawn from the mice via retro-orbital bleeding and plasma separated by centrifugation. Plasma was spiked with BP and analyzed for CA and CL by LC–MS/MS as described above (Section 2.4.). Pharmacokinetic modeling was performing using Phoenix WinNonLin 8.1.

### 2.6. Immunofluorescence of ZO-1 in HT-29 Cells

ZO-1 proteins were analyzed by immunofluorescence microscopy following our previously described protocol [41]. HT-29 cells were seeded in a Millicell EZ 8-well chamber slides (Cat. PEZGS0816, Millipore Sigma) and allowed to differentiate for 6 days [42]. Cells were fixed in paraformaldehyde solution, permeabilized with 0.2% TritonX-100, and blocked with 5% bovine serum albumin (BSA). Then, slides were incubated with rabbit anti-ZO-1 primary antibody (1:200) followed by incubation with Alexa Fluor 488-conjugated goat anti-rabbit IgG (H + L) secondary antibody (Cat. 111-545-144, Jackson ImmunoResearch, PA, USA) at room temperature. Slides were washed with phosphate buffered saline (PBS) and mounted with Prolong Gold Antifade with 4′6-diamidino-2-phenylindole (DAPI) (Cat. 8961S, CST) and allowed to dry for 24 h. Microscopy was performed on a Zeiss LSM 710 Meta Confocal Laser Scanning Microscope (Carl Zeiss AG, Oberkochen, Germany). Image processing and intensity quantification were performed using ZEN 2011 (blue edition) software. Since TJ expression is dependent on the number of cells, the fluorescence intensity of the image area was divided by the number of cells shown [43].

### 2.7. Rosemary Extract Dosing and Colitis Induction

The preventive effects of RE against dextran sodium sulfate (DSS)-induced colitis was tested in mice (permission number 19-075). Oil-soluble RE was dissolved in cottonseed oil and administered by oral gavage daily to mice at 10 and 100 mg/kg/day (HED = 0.81 and 8.1 mg/kg, respectively) for 10 consecutive days. Beginning on day 3 and continuing through day 10, mice were given 3.5% DSS in the drinking water to induce colitis. At the conclusion of the experiment, mice in each group were evaluated for disease activity index (DAI) on the basis of percent body weight change, fecal consistency, and fecal blood [44,45]. The scoring for each parameter was as follows: body weight change: 0 = 0–1%, 1 = 1–5%, 2 = 6–10%, 3 = 11–20%, 4 = >20%; fecal consistency: 0 = normal pellets, 2 = loose stool, 4 = diarrhea; fecal blood: 0 = no apparent blood, 2 = some blood in stool, 4 = severe blood.

### 2.8. Tissue Collection

At the completion of the study, colons from euthanized mice were taken from the cecum to the distal colon. The colon was then washed in ice-cold PBS to remove any remaining fecal matter and weighed. The colon was divided in half, with one portion stored in RNAlater solution and the other in PBS for protein analysis. The spleen was also removed and weighed (Table S1). Tissues were stored at −80 °C until analysis.

### 2.9. Quantitative RT-PCR

Total RNA was extracted from intestinal tissue using the RNeasy PowerLyzer Tissue and Cells kit from Qiagen (Germantown, MD, USA), according to the manufacturer’s instructions. Then, 2 µg RNA was reverse-transcribed to complementary DNA (cDNA) using the OneStep RT-PCR kit (Life Technologies, Grand Island, NY, USA) in a Bio-Rad C1000 Thermal Cycler (Bio-Rad, Hercules, CA, USA). Quantitative PCR was performed using QuantiFast SYBR Green PCR kit (Qiagen, Germantown, MD, USA) in a StepOne Real-Time PCR System (Thermo Fischer Scientific, Waltham, MA, USA). GAPDH was used as control for normalization. Primers were obtained from Integrated DNA Technologies (Coralville, IA, USA), and sequences are as follows: Sesn2, forward: 5′-GAGCTGGAGAAGTCAGAAAG-3′, reverse: 3′-GGTCCTCCACAAAGCATAG-5′.

### 2.10. Western Blot Analysis

Total protein content isolated using the RNeasy PowerLyzer Tissue and Cells kit (see Section 2.10) was quantified using the 2-D Quant Kit from GE Healthcare (Pittsburgh, PA, USA) according to the manufacturer’s instructions. Western blot analysis was performed as previously described [46]. Denatured proteins were subjected to electrophoresis in a 7.5–15% gel. Proteins transferred to a nitrocellulose membrane and incubated with primary antibodies (1:1000) overnight. Membranes were then incubated in secondary antibody (1:2000) for 2 h and exposed in a Cell Biosciences FluorChem E imager (Santa Clara, CA, USA). ImageJ software was used to quantify expression.

### 2.11. FITC–Dextran Analysis

On the final day of the experiment, mice were administered FITC–dextran (40 kDa) 4 h before euthanization to assess intestinal barrier integrity. Immediately before euthanization, blood was collected via retro-orbital bleeding. Plasma was separated by centrifugation at 1500× *g* for 15 min, and 200 µL of each sample was added to a 96-well black microplate. Fluorescence was read with a Biotek Synergy HT plate reader (Agilent Technologies, Santa Clara, CA, USA) at 495/520 nm wavelength.

### 2.12. Statistical Analysis

Data are represented as mean ± standard error of the mean. Data were analyzed by one-way ANOVA followed by Tukey’s honestly significant difference (HSD) test. A *p*-value < 0.05 was considered statistically significant.

## 3. Results

### 3.1. Identification of Major Components in Rosemary Extract

**Figure 1:**
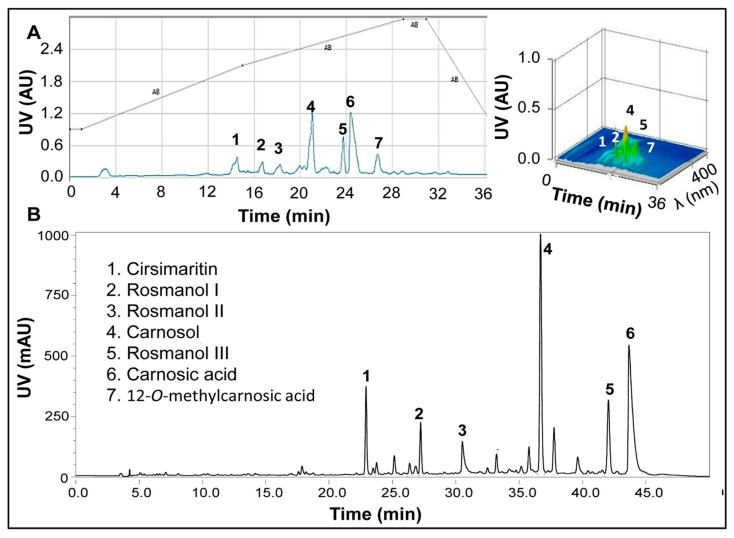
Separation and identification of major compounds in oil-soluble rosemary extract (RE). (**A**) Representative semi-preparative HPLC chromatogram of RE along with the corresponding 3D UV scan. (**B**) Representative analytical HPLC chromatogram of RE. Details of semi-preparative and analytical methods are described in Section 2.2. Peaks were identified by LC–MS/MS.

Phytochemicals in RE were separated by HPLC and identified by LC–MS analysis. Identities of the major peaks (Figure 1) were confirmed by interpretation and comparing mass spectra with those of previous reports [37,38,39]. All compounds have previously been reported and were identified as cirsimaritin (1), rosmanol I (2), rosmanol II (3), CL (4), rosmanol III (5), and CA (6) (Figure 1). Peak 5 (Figure 1B) is an unknown compound. Isolation of peak 5 was not pursued because it appeared to be a broad and poorly separated peak in the semi-preparative method (Figure 1A). Peak 7 was only detected in the semi-preparative extraction and was identified as 12-O-methylcarnosic acid.

### 3.2. Pharmacokinetic Analysis of Oil-Soluble Rosemary Extract

An LC–MS/MS method was developed to accurately quantify CL and CA concentrations in mouse plasma. The lower limit of detection (LLOD) of CL was 5 ng/mL, and the lower limit of quantification (LLOQ) was 10 ng/mL. The LLOD and LLOQ of CA were 10 and 250 ng/mL, respectively.

**Figure 2:**
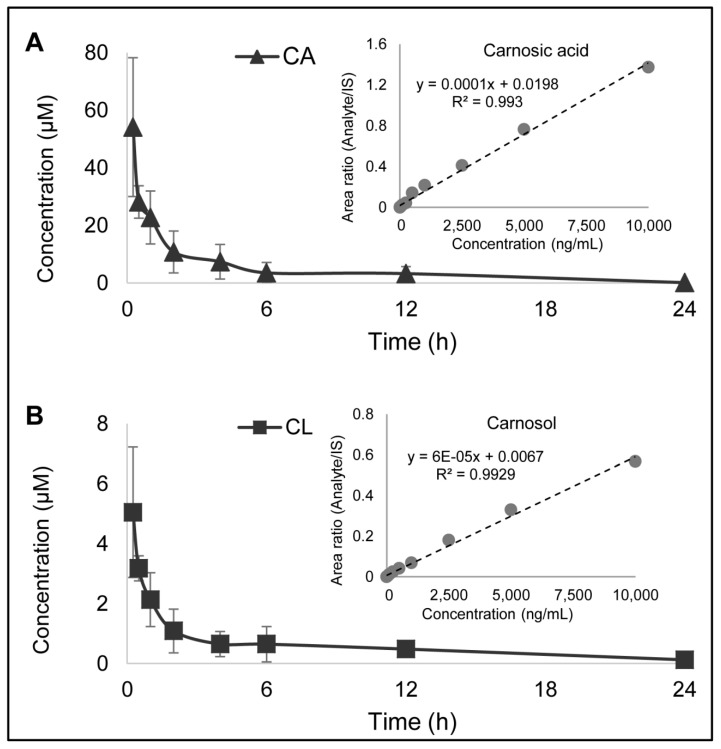
Concentration curve of the pharmacokinetic profile of (**A**) carnosic acid and (**B**) carnosol when administered as rosemary extract (RE) in mice. Mice were given 100 mg/kg of oil-soluble RE by oral gavage, and blood was drawn at time points of 0.25, 0.5, 1, 2, 4, 6, 12, and 24 h. The plasma was separated and analyzed by LC–MS/MS. The pharmacokinetic parameters determined from the study are found in Table 1.

The pharmacokinetic profile of CL and CA from oil-soluble RE was determined (Figure 2). On the basis our analysis and vendor analysis per the certificate of analysis, we found that the RE contained 40% (*w*/*w*) CA and 5% CL. Mice were administered 100 mg/kg RE, and blood was drawn at pre-determined time points over 24 h. CA and CL were detected simultaneously by LC–MS/MS as described above. As anticipated, plasma contained a significantly higher concentrations of CA compared to CL. The maximum concentration (Cmax) for CA was 54.016 µM at 0.25 h post-dosing, and the half-life was 3.5 h. The Cmax for CL was 5.008 µM at 0.25 h post-dosing, and the half-life was 7.5 h (Table 1).

### 3.3. Rosemary Extract Protected against tBHP-Induced Disruption of ZO-1 Protein Expression

**Figure 3:**
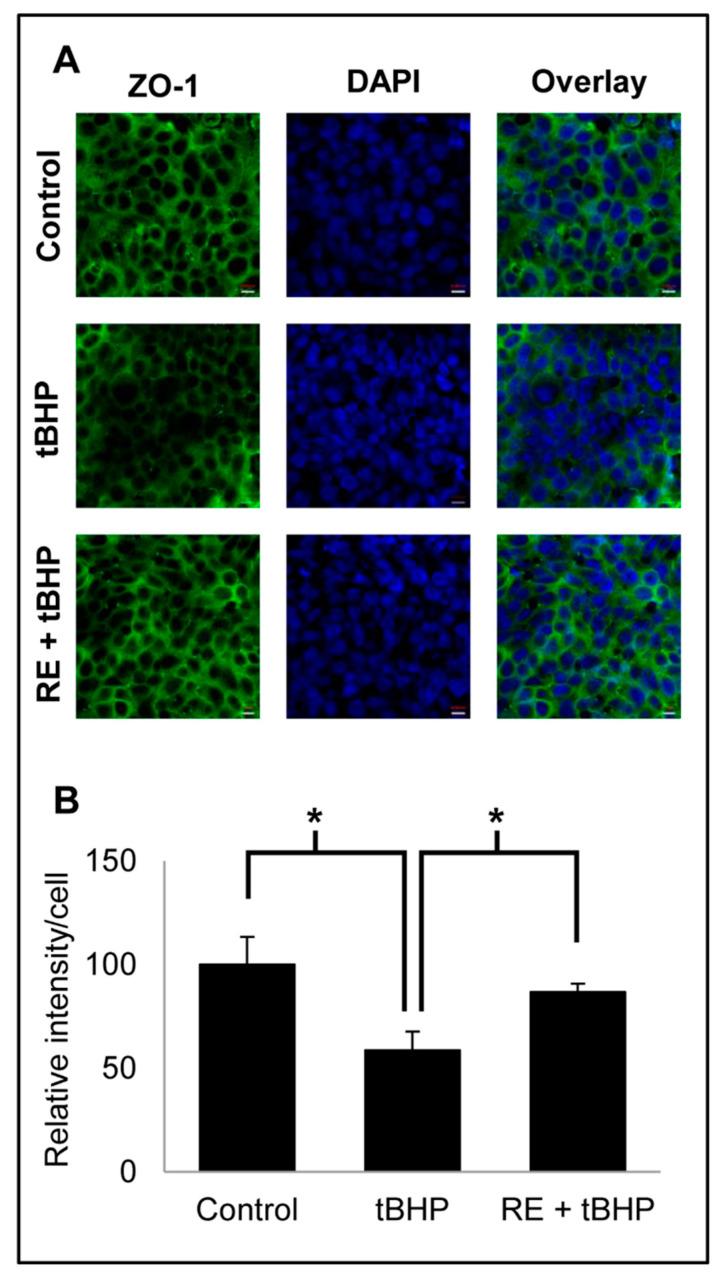
(**A**) Immunofluorescence images (magnification 63×/1.46 oil) showing the protective effect of rosemary extract against tert-butyl hydroperoxide (tBHP)-induced zonula occludens-1 (ZO-1) protein loss. Cells were pretreated without or with 20 µg/mL rosemary extract for 24 h, followed by exposure to 100 µM tBHP for 16 h. Immunofluorescence protocol (described in Section 2.6.) was followed to probe for ZO-1 proteins using a confocal microscope. (**B**) Immunofluorescence data were expressed as fluorescence intensity per cell relative to the control. One-way ANOVA followed by Tukey’s honestly significant difference (HSD) test were used to determine significance. * *p* < 0.05.

The protective effect of RE on the epithelial TJ was demonstrated by confocal microscopy of ZO-1 protein in HT-29 cells (Figure 3). Exposure to non-toxic levels (50 µM, 16 h) of tert-butyl hydroperoxide (tBHP), a commonly used chemical agent to induce cellular oxidative stress, reduced ZO-1 fluorescence intensity to 58% of the control, but a 24-h pre-treatment with RE (20 µg/mL) and subsequent co-incubation with tBHP for 16 h rescued 67% of ZO-1 intensity lost to tBHP treatment.

### 3.4. Rosemary Extract Improved DAI in DSS Colitis Mice

**Figure 4:**
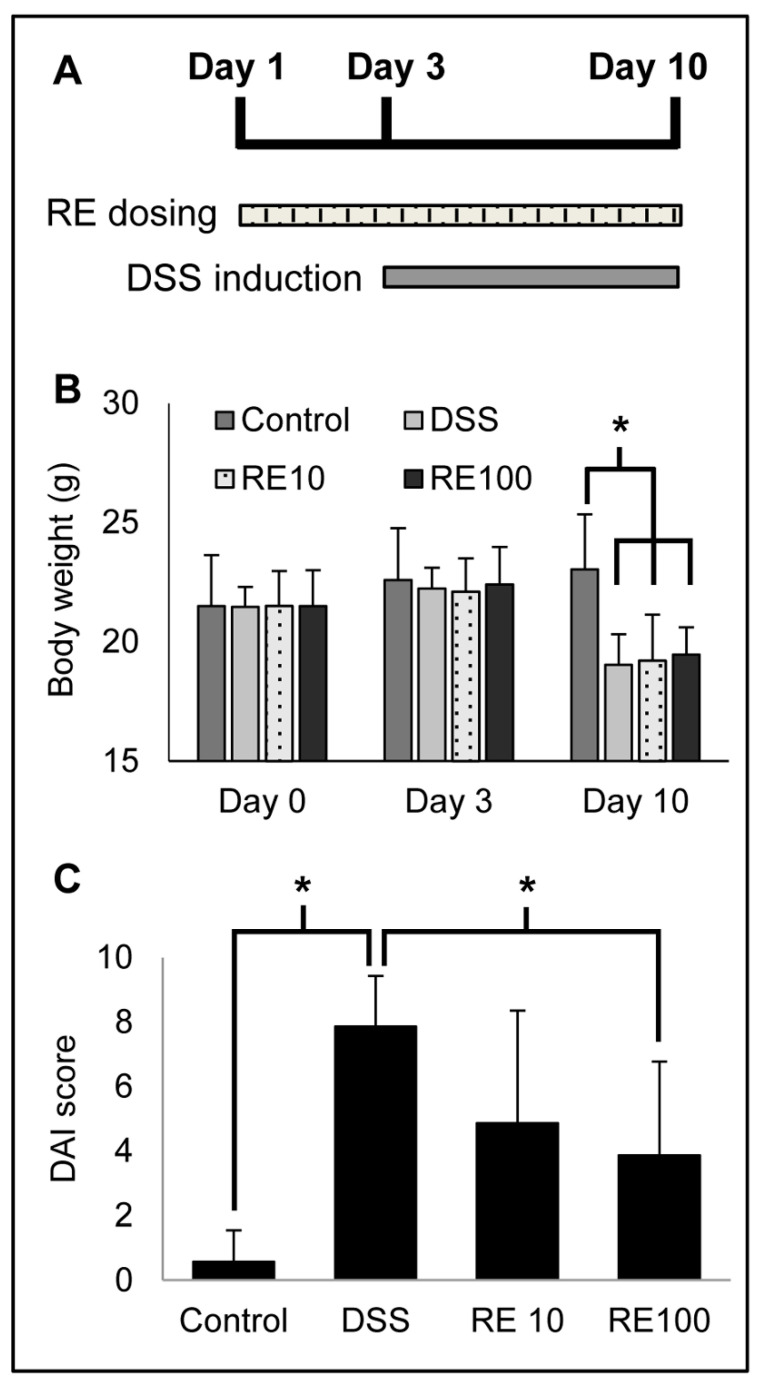
(**A**) Visual timeline of in vivo study. Mice were pre-treated with rosemary extract (RE) for 3 days prior to dextran sodium sulfate (DSS) administration for 7 days. Seven mice were included in each group. (**B**) Disease activity index (DAI) scores for DSS mice treated with RE (*n* = 7 mice per group). Mice were scored according to parameters associated with colitis injury. The results show pre-treatment with RE100 improved DAI scores compared to DSS. (**C**) Body weight changes in DSS mice treated with RE (*n* = 7 mice per group). Mice were weighed on day 0 (before RE administration), day 3 (before DSS administration), and day 10 (before sacrifice). One-way ANOVA followed by Tukey’s HSD test was used to determine significance. * *p* < 0.05.

**Figure 5:**
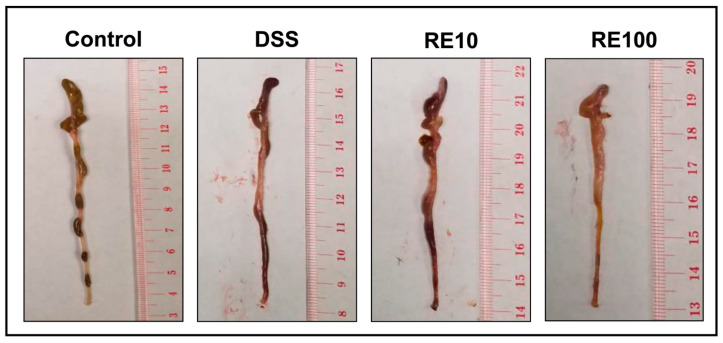
Representative images of mouse colons from each group (*n* = 7 mice per group): Control, DSS, RE10, and RE100. Colons were excised from the cecum (top) to the distal colon (bottom) and washed in ice-cold phosphate buffered saline (PBS). Cleaned colons were then measured, weighed, and stored in ice-cold PBS at −80 °C. DSS causes shortening and bleeding in the intestine along with increased diarrhea to the mouse. Visually, the colons from the DSS and RE10 mice contained higher amounts of blood compared to the control, but this bleeding appeared to be suppressed in the RE100 mice, indicating lower disease activity from DSS administration.

An in vivo experiment of DSS-induced colitis was used to determine the preventive effect of RE pre-treatment. The timeline of the experiment is shown in Figure 4A and is outlined above. The disease activity index (DAI) for each mouse was used to quantify the efficacy of rosemary pre-treatment for colitis prevention. The DAI was scored on the basis of percent change in body weight, fecal consistency, and fecal blood, as detailed in Section 2.7. The body weights of the mice are shown in Figure 4B, as DSS causes body weight reduction. The DAI parameters were combined into a single score for each mouse, and the individual scores were averaged for the respective groups (*n* = 7 mice per group) (Figure 4C). The individual parameters and DAI scores are in Table 2. Images of mouse colons were taken and revealed a significant reduction in bleeding in the RE100 group (Figure 5).

The analysis revealed RE at 100 mg/kg protected against DSS-induced colitis as shown by improved DAI (i.e., lower score) compared to the DSS group (i.e., vehicle-control group). Particularly, rosemary pre-treatment improved the fecal consistency (solid pellets) and contained less fecal blood compared to DSS mice. These results suggest that RE protected against DSS to reduce inflammation and bleeding.

### 3.5. Rosemary Extract Prevented Increased Sestrin 2 Protein Expression

**Figure 6:**
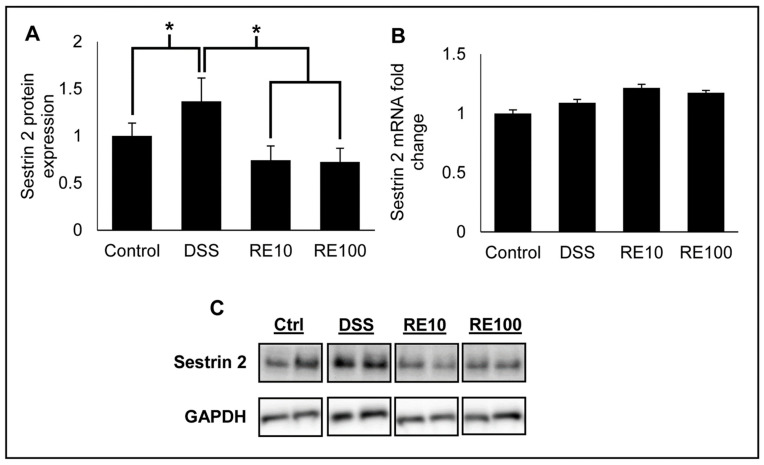
(**A**) Rosemary extract (RE) protected against increased sestrin 2 protein expression caused by DSS. Mouse intestinal lysates were analyzed for sestrin 2 protein expression by Western blot (*n* = 7 samples per group). (**B**) Sestrin 2 mRNA analysis by qPCR showed that sestrin 2 mRNA was not significantly affected by DSS exposure or with RE pre-treatment. (**C**) Western blot images of sestrin 2 protein expression in mouse colon lysates representative of each group from the DSS colitis experiment. One-way ANOVA followed by Tukey’s HSD was used to determine significance. * *p* < 0.05.

Reports have suggested that loss of sestrin 2 correlates with severe colitis due to its role in maintaining intestinal homeostasis [33]. Therefore, the effect of RE pre-treatment on expression of sestrin 2 protein in DSS colitis mice was determined. Lysates from mouse intestinal tissues were prepared according to Section 2.8, and sestrin 2 protein expression was measured by Western blot (Figure 6A,C). The results revealed that DSS exposure increased sestrin 2 protein expression, likely due to increased cellular stress caused by DSS. Pre-treatment with RE at both 10 and 100 mg/kg suppressed sestrin 2 upregulation, indicating that RE protects against DSS-induced cellular stress. Interestingly, there was not a significant change in sestrin 2 mRNA (Figure 6B), which could suggest that the phytochemicals in RE regulate sestrin 2 expression post-transcriptionally.

### 3.6. Rosemary Prevented Loss of Intestinal Barrier Integrity

**Figure 7:**
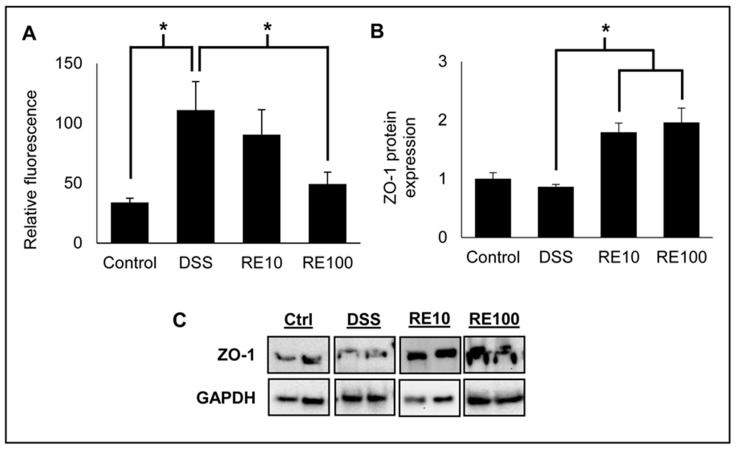
Rosemary extract (RE) protected against loss of barrier integrity in DSS-induced colitis mice. FITC–dextran was administered and analyzed in mouse plasma according to Section 2.12 (*n* = 7 samples per group). (**A**) DSS administration significantly increased plasma levels of FITC, indicating severe loss of intestinal barrier integrity. RE at 100 mg/kg protected intestinal barrier disruption as shown by decreased plasma FITC compared to negative control. (**B**) Zonula occludens-1 (ZO-1) protein expression from mouse colon lysates was analyzed by Western blot. RE administration at both 10 and 100 mg/kg significantly increased ZO-1 protein expression. (**C**) Western blot images of ZO-1 protein expression in mouse colon lysates representative of each group from the DSS colitis experiment. One-way ANOVA followed by Tukey’s HSD was used to determine significance. * *p* < 0.05.

The integrity of the intestinal TJ barrier was evaluated by fluorescence spectroscopy of FITC–dextran in mouse plasma (Figure 7A). FITC (40 kDa) was administered to mice orally, and blood was collected 4 h following administration of RE. The fluorescence in the plasma was quantified to determine the amount of FITC that passed through the intestinal barrier and into the bloodstream. Mice with DSS exposure had significantly increased FITC levels, indicating disruption of the intercellular TJs consistent with DSS exposure. Rosemary pre-treatment at 100 mg/kg revealed an improvement in barrier integrity compared to DSS-only mice. These results indicate that RE protects against damage caused to the intestinal TJ barrier by DSS exposure, which is consistent with the DAI assessment. Furthermore, Western blot analysis revealed that the expression of ZO-1 was significantly increased in both treatment groups (Figure 7B,C). ZO-1 expression is associated with improved epithelial barrier function, suggesting that RE prevents colitis induction through modulating TJ protein expression [47].

## 4. Discussion

In summary, the pharmacokinetics of CA and CL, two major diterpene constituents in rosemary, and the ability to prevent colitis development using oil-soluble RE containing CA and CL in mice was determined. A dose of 100 mg/kg of RE was found to decrease the DAI compared to DSS-treated mice, suggesting that development of colitis was prevented. Sestrin 2 protein was lower in the rosemary-treated groups compared to the DSS group, suggesting a decrease in cellular stress of the cells within the colon. Analysis of FITC levels in mouse plasma were also reduced in rosemary treatment groups, suggesting that RE is beneficial in maintaining the intestinal barrier integrity, which is often compromised in colitis.

LC–MS/MS of rosemary extracted with methanol revealed eight major phytochemicals, seven of which were identified. These compounds all belong to the phenolic diterpene class of compounds known for their antioxidant activity. Our group as well as others has shown that phenolic diterpenes, including CA and CL, have therapeutic potential for diseases such as cancer, diabetes, and colitis [48,49,50,51]. Reports have suggested that CA and CL can contribute up to 90% of the antioxidant activity in RE, making them particularly interesting for further studies [52]. Therefore, we decided to investigate the pharmacokinetics of CA and CL in mice while administered as a RE. The Cmax of CA was nearly 11-fold higher than that of CL in mice dosed with 100 mg/kg RE, which is expected since the amount of CA in the extract is eightfold higher than CL. However, the half-life of CA was found to be much shorter than CL, being 3.5 h compared to 7.5 h, respectively.

Previously, our lab has shown that CL from RE activates the Nrf2 pathway to modulate sestrin 2 expression. In this study, RE treatment at both 10 and 100 mg/kg doses prevented increase in sestrin 2 protein levels seen in the DSS group. An interesting observation is that sestrin 2 expression, which is typically considered beneficial, was increased with DSS exposure in our study. This observation is contrary to many studies in cancer and diabetes, where sestrin 2 is decreased and elevating the expression improves the condition of these disease states [53,54]. However, in this model of acute colitis, DSS exposure increased sestrin 2 expression while co-treatment with RE normalized the expression pattern of sestrin 2. This hypothesis is supported by earlier studies that have reported that sestrin 2 protein expression is increased in patients with ulcerative colitis [33]. Interestingly, some reports have suggested that sestrin 2 expression can be elevated in some cancers, such as the non-small cell lung cancer (NSCLC) line A549, and that its expression is negatively correlated with lung cancer patient survival [55]. Therefore, sestrin 2 can be viewed as having a biphasic response depending on the disease state that is being observed, which could determine the ability of sestrin 2 being a therapeutic target in certain conditions. However, the role of sestrin 2 in IBD development and disease state is unclear, and therefore more research is needed to determine the effects of sestrin 2 protein modulation in the context of IBD. On the basis of our previous results, we believe that the mechanism of action of diterpenes from RE is directed primarily through the Nrf2 pathway [19]. Several studies have shown that activating Nrf2 is beneficial for preventing colitis development [56,57,58].

Intestinal permeability is an important contributing factor to the pathogenesis of IBD, and therefore our study sought to determine the impact of RE on intestinal barrier integrity and TJ protein expression [59,60]. Analysis of FITC revealed that RE at 100 mg/kg reduced the intestinal permeability compared to negative control mice, and Western blot revealed upregulation of ZO-1 protein expression with RE treatment. The increase in barrier integrity correlated with the improvement in DAI, suggesting that modulation of TJs is a key mechanism for RE-mediated prevention of colitis. The expression of ZO-1, an important peripheral membrane TJ protein, was significantly decreased in the negative control group. This result is in agreement with several studies that show loss of ZO-1 leads to disrupted barrier function [61,62]. However, RE pre-treatment prevented ZO-1 downregulation, thereby lowering intestinal permeability caused by DSS exposure.

Future studies can be aimed at elucidating a thorough mechanism for RE prevention of colitis. This study suggests that improved TJ barrier integrity plays a key role in colitis prevention. Treatment with RE could be strengthening the intestinal barrier for protection against chemical irritants such as DSS, but further studies are needed to confirm this hypothesis.

## 5. Conclusions

This study is significant because it demonstrated that RE has the ability to prevent colitis development in vivo. RE was also shown to improve intestinal barrier integrity, which correlated with reduction in DAI. This study also has clinical relevance because sestrin 2 protein expression, which is elevated in patients with ulcerative colitis, was reduced by RE administration. This research provides further evidence that rosemary can provide GI health benefits in addition to food preservation.

## Figures and Tables

**Table 1:** Pharmacokinetic parameters of carnosic acid and carnosol from rosemary extract (100 mg/kg).

Compound	Half-Life (h)	Tmax (h)	Cmax (µM)	AUC (µM × h/mL)
Carnosic acid	3.5	0.25	54.016	119.7
Carnosol	7.5	0.25	5.008	14.0

**Table 2:** Disease activity index (DAI) scoring for DSS colitis mice treated with RE. Scoring is based on parameters defined in Section 2.7 (* *p* < 0.05 compared to DSS).

Group	Body Weight Percent Change †		Fecal Blood		Fecal Consistency		DAI Score	
	Mean	SD	Mean	SD	Mean	SD	Mean	SD
Control	0.71	0.20	0.0	0.0	0.57	0.98	1.29	0.47
DSS	2.86	0.15	3.43	1.15	2.00	0.0	8.29	1.50
RE10	2.86	0.37	1.71 *	2.14	0.86 *	1.07	5.43	1.30
RE100	2.71	0.20	1.14 *	1.95	0.57 *	0.98	2.70 *	1.02

† Body weight percent change is calculated on the basis of the final weight of the mouse compared to the weight on day 0.

## Data Availability

The data presented in this study are available on request from the corresponding author.

## Supplementary Materials

The following are available online at https://www.mdpi.com/2072-6643/13/3/773/s1: Table S1: Average colon and spleen weight of DSS mice treated with RE (*n* = 7 mice per group).
