# Supplementary material for: Pharmacokinetic Analysis of Carnosic Acid and Carnosol in Standardized Rosemary Extract and the Effect on the Disease Activity Index of DSS-Induced Colitis
Source: Nutrients. 2021 Feb 27;13(3):773. doi: 10.3390/nu13030773 (PMC7997407; doi:10.3390/nu13030773)
Supplement: Supplementary file 1 [file nutrients-13-00773-s001.pdf]

Table S1. Average weights of colons and spleens from DSS-mice treated with RE (n=7 mice per group).

Group	Colon weight (g)		Spleen weight (g)	
	Mean	St dev	Mean	St dev
Control	0.49	0.067	0.08	0.009
Colitis	0.46	0.065	0.10	0.018
RE10	0.45	0.080	0.11	0.026
RE100	0.48	0.042	0.10	0.021
